# Incidence and outcome of patients suffering from meningitis due to spondylodiscitis

**DOI:** 10.1016/j.bas.2023.101781

**Published:** 2023-08-25

**Authors:** Insa K. Janssen, Yu-Mi Ryang, Maria Wostrack, Ehab Shiban, Bernhard Meyer

**Affiliations:** a Department of Neurosurgery, Hôpitaux Universitaires Genève, Rue Gabrielle-Perret Gentil 4, 1205, Geneva, Switzerland; b Department of Neurosurgery, Klinikum rechts der Isar, Technical University Munich, Ismaninger Straße 22, 81675, Munich, Germany; c Department of Neurosurgery, Helios Klinikum Berlin-Buch, Schwanebecker Chaussee 50, 13125, Berlin, Germany; d Department of Neurosurgery, Universitätsklinikum Augsburg, Stenglinstr. 2, 86156, Augsburg, Germany

**Keywords:** Bacterial meningitis, Epidural abscess, Neck stiffness, Spondylodiscitis, Confusion, Pyogenic spinal infection

## Abstract

**Introduction:**

Meningitis is a rare but severe complication in patients with spondylodiscitis. Data about the incidence and clinical management are rare.

**Research question:**

Aim of this study was to assess the incidence, clinical course and outcome of patients suffering from meningitis due to spondylodiscitis.

**Material and methods:**

We performed a retrospective analysis of our prospective clinical database that included all patients suffering from spondylodiscitis between January 2010 and December 2019 were included. We assessed clinical findings, laboratory tests, treatment and outcome comparing patients with and without meningitis.

**Results:**

Out of 469 patients suffering from spondylodiscitis, 30 patients (14 female) were diagnosed with an associated meningitis (6.4%). The mean CSF cell count was 3375.85 ± 8486.78/μl (range 32-41500/μl). The mean age at presentation was 70.87 ± 8.84 yrs (range 48-88 yrs). Mean C-reactive protein (CRP) and white blood cell (WBC) counts at time of admission were statistically higher in patients with associated meningitis (CRP: 19.81 ± 12.56 mg/dl vs. 11.63 ± 11.08 mg/dl, p = 0.001; WBC: 14.67 ± 7.76 g/l vs. 10.88 ± 05.11 g/l, p = 0.005. Mortality was also higher, as 13.3% and 7.1% of patients with and without concomitant meningitis died, respectively.

**Conclusion:**

Bacterial meningitis due to spondylodiscitis is a rare but severe condition and is associated with higher morbidity and mortality rates. In patients with spondylodiscitis presenting with an altered state of consciousness an associated meningitis should be ruled out.

## Highlights

• The incidence of bacterial meningitis due to spondylodiscitis is low but associated with high morbidity and mortality rates.
• Diagnosis of spondylodiscitis may be delayed due to nonspecific symptoms especially in elderly patients.
• Most frequent causative organism is *Staphylococcus aureus.*

## 1 Introduction

Bacterial meningitis is a rare but severe complication of patients with spondylodiscitis (Ohnishi et al., 2015; Tali et al., 2015). The incidence of spondylodiscitis ranges from 0.2 to 2.4 per 100,000 people per year in western countries (Gouliouris et al., 2010; Cheung and Luk, 2012) and is a serious disease associated with a significant morbidity and mortality rate ranging between 2 and 20% (Zarghooni et al., 2012; Skaf et al., 2010). Mortality results in most cases from associated systemic complications rather than local infection. Septic distribution through spread into the blood stream (bacteraemia) or cerebrospinal fluid (meningitis) may result in multiorgan failure and shock (Heuer et al., 2022). Although bacterial infection of the central nervous system (CNS) is a life-threatening neurological emergency (Roos, 2015; Kohlmann et al., 2015; Hussein and Shafran, 2000), only few papers report on single cases of meningitis, e.g., due to epidural abscess after epidural neuroplasty (Lee et al., 2015), or in the case of a panspinal epidural abscess in an 41-year-old man with diabetes (Lin et al., 2013). In contrast, there are several studies report on community-acquired bacterial meningitis (CABM). The mortality rate of CABM is about 21% and varies depending on the causative organism (van de Beek et al., 2006). Bacterial meningitis has a classic symptomatic triad of fever, neck stiffness and a change in mental status characteristic to it. However, only 44% of patients show the characteristic classic triad in a study by van de Beek et al. (van de Beek et al., 2004). Many patients with bacterial meningitis present with an abnormal state of consciousness, and 15–20% of patients are comatose upon presentation (van de Beek et al., 2004). Bacterial meningitis can cause complications like hydrocephalus, cranial empyema, cerebral (micro-)infarctions or epileptic seizures (van de Beek et al., 2006). The method of choice for diagnosis of bacterial meningitis is the analysis of cerebral spinal fluid (CSF) (Grindborg et al., 2015; Troendle and Willis, 2017). Predisposing conditions for bacterial meningitis, epidural empyema and spondylodiscitis are medical conditions resulting in immunodeficiency like splenectomy, infectious diseases, diabetes mellitus, cancer or intravenous drug abuse (Adriani et al., 2015; Arko et al., 2014). Risk factors include impaired mental status, older age, comorbidity, and fulminant disease, and delayed antibiotic treatment constitutes a major risk factor for a poor outcome (Grindborg et al., 2015; van de Beek et al., 2012). Furthermore, CRP is an important independent prognostic information in septic patients (Heuer et al., 2022).

Antibiotic treatment, according to international guidelines, includes intravenous treatment between 7 and 21 days, depending on the germ causing the disease and the individual response of the patient (van de Beek et al., 2016).

Detection of spinal infections can be challenging due to non-specific symptoms, such as back pain and fever, and diagnosis is verified by MRI (Bangstrup et al., 2016). Despite its increasing incidence (Dobson et al., 2015), treatment of spondylodiscitis still remains controversial, particularly due to a lack of randomized controlled trials and prospective studies (Quack et al., 2014). Established treatment of patients suffering from uncomplicated spondylodiscitis is still a conservative management regime (Bernard et al., 2015), consisting of long-time antibiotic therapy (Bernard et al., 2015) optionally combined with immobilization by bed rest or an orthosis (Bangstrup et al., 2016). In cases of neurological deficits, epidural abscess and spinal instability or failure surgical, treatment with or without instrumentation of the spine is performed. If there is relevant bone loss due to the infection, iliac crest autographs or titanium cages are generally used to replace the vertebra. Some authors have recently described antibiotic-loaded PMMA (polymethyl methacrylate) cement for anterior column reconstruction after debridement as a good, safe option (Banse et al., 2022; Deml et al., 2022). Spinal epidural abscess in general has an incidence of about 2 to 25 patients per 100,000 admitted to the hospital (Al-Hourani et al., 2016). Most patients with spinal epidural abscess classically present with fever, low back pain and focal neurological deficit (Lin et al., 2013).

The aim of this retrospective monocentric study was to establish a general idea of the incidence of associated meningitis due to spondylodiscitis and to evaluate the clinical course and outcome of these patients, expecting a higher morbidity and mortality compared to patients suffering from spondylodiscitis without associated meningitis.

## 2 Methods

We performed a retrospective analysis of our clinical database. All patients suffering from spondylodiscitis between January 2010 and December 2019 were included, focusing on patients with a suspicion of an associated meningitis. We assessed clinical findings, laboratory tests, treatment and outcome. Diagnosis of meningitis was confirmed by a CSF examination. We estimated the CSF cell count as well as the amount of protein, glucose and lactate. Furthermore, C- reactive protein (CRP) and white blood cells (WBC) were considered. In order to prevent additional introduction of infection into the CSF and also not to falsify the results, the MRI images were carefully analysed before the lumbar puncture was performed. Whenever possible, the lumbar puncture was performed at a level unaffected or least affected by the spondylodiscitis. A puncture through a paravertebral infiltration as well as through an epidural abscess was strictly avoided.

The study was approved by an ethics committee (No.238/17 S). Patient consent was not necessary because all patients signed the consent of the clinic for storage and analysis of tissue and blood samples, etc., for the purposes of science.

### 2.1 Statistical analysis

Statistical analysis was performed using IBM SPSS Statistics Version 24.0 (SPSS Inc., IBM Corp., Armonk, NY, USA). Normally distributed variables are shown as the mean and standard deviation. A *t*-test was used to compare laboratory tests and the duration of hospital stays for both patient groups. A p value < 0.5 was assumed to be statistically significant.

## 3 Results

**Fig. 1:**
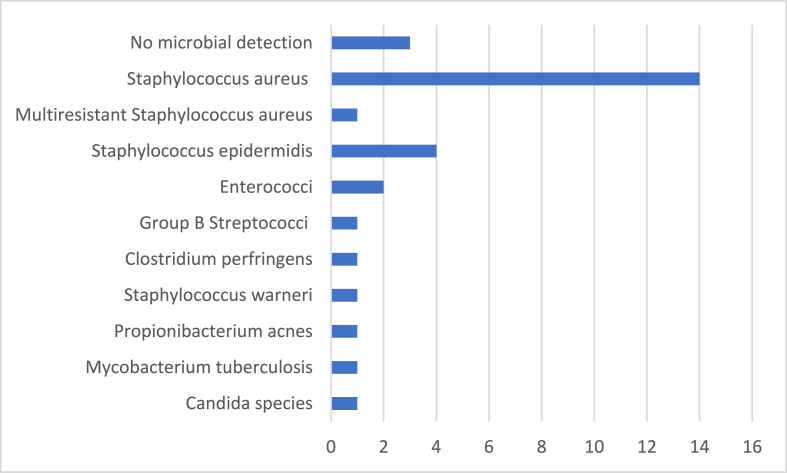
Causative microbial organism in patients with associated meningitis (n = 30).

Out of 469 patients suffering from spondylodiscitis between January 2010 and December 2019, 30 patients (6.4%) were diagnosed with associated meningitis (see demographics, Table 1). The mean age at admission of patients with meningitis was 70.87 ± 8.84 years (range 48-88 years). Sixteen patients (53.33%) initially presented with neurological deficits; two of them were confused. Two further patients required ICU treatment with ventilation at the time; 12 patients (40%) were neurologically intact, but two of them showed a change of consciousness. All patients were diagnosed for spondylodiscitis (for localization, see demographics Table 1), assumed foci are listed in Table 2. Diagnosis of meningitis was confirmed by a CSF examination. The mean CSF cell count at diagnosis was 3375.85/μl ± 8486.78/μl (range 32-41500/μl; Table 1). CSF samples were not routinely taken from all spondylodiscitis patients. The most frequent reason for performing an CSF examination was the suspicion of meningitis due to delirium, confusion or worsening of consciousness (n = 22; 73.33%). Three suffered an epileptic seizure. In four patients, the apparition of a new neurological deficit, e.g., aphasia and hemiparesis, led to a CSF investigation. Only five (16.67%) showed stiffness of the neck; ten (33.33%) were suffering from additional fever. None showed the classic triad of fever, neck stiffness and change in mental status characteristic for bacterial meningitis. In the other cases, the CSF was gained to conduct a (CT)-Myelography to rule out a myelitis (n = 1) and after the lumbar drain placement due to a cervical CSF leak resulting from an inflammatory eroded dura mater (n = 3). In these cases, meningitis was diagnosed by chance. In 21 patients, cerebral imaging for additional diagnostics was performed (cerebral CT scan, n = 6; cerebral MRI, n = 15). In two cases, purulent accumulation in the posterior horn of the ventricle was present. An acute or subacute ischemic area due to septic cerebral embolism or vasculitis was observed in four patients, in one case, accompanied by limbic encephalitis. Increased gadolinium enhancement of the meninges as a radiological sign of meningitis was seen in three patients. Eighteen (60%) patients required ICU treatment for an average of 19.39 ± 23.06 days (range 1-94). Long-term ventilation and, therefore, tracheotomy was necessary in four cases. The placement of an external ventricular drainage was performed in five patients because of hydrocephalus (n = 1) or for neuromonitoring after intubation (n = 4). The mean time to diagnose meningitis after admission to our department was 8 days ±8.32 days, whereas antibiotic treatment had already been introduced to treat spondylodiscitis. Successful isolation of the microbial organisms was possible in 27 (90%) cases. The most frequent organisms were *Staphylococcus aureus* in 15 patients (50%), one a multi-resistant *Staphylococcus aureus* (MRSA), and Staphylococcus epidermidis in four patients (13.33%; Fig. 1. In the majority of cases, the microbial organism was detected through intraoperative or CT guided biopsy (n = 20) and/or blood culture (n = 15). In nine cases, the same pathogens could also be isolated in the CSF. To treat underlying spine infections, 29 patients received surgical instrumentation of the spine, followed by 20.07 ± 8.58 (range 14-46) days of intravenous antibiotic therapy. An oral antibiotic therapy was administered then for another 10.78 ± 1.57 (range 10-16) weeks. One patient was treated conservatively because of a very poor general health condition, and one patient refused surgical treatment. The mortality rate in this group of patients suffering from associated meningitis was 13.33% (n = 4). One patient experienced important cerebral infarction either as complication due to bacterial vasculitis or because of septic embolism. She also suffered from epileptic seizures and ultimately died due to multi-organ failure. An 85-year-old female suffering from spondylodiscitis and meningitis by mycobacterium tuberculosis was transferred to the palliative care unit after she became comatose and showed no improvement in her neurological status after a few weeks of treatment. At the time of transfer to the rehabilitation centre eleven patients (36.67%) were still impaired by neurological deficits, and 15 (50%) were intact. Both, the mean CRP value and mean WBC count declined significantly in all cases upon discharge (Table 1). Comparing with patients without diagnosed meningitis (n = 439), there was no statistically significant difference regarding the mean age at presentation between both groups (70.79 ± 8.98 vs 67.9 ± 13.1, p = 0.490). In contrast, the mean CRP and WBC counts at the time of admission were statistically higher in patients with associated meningitis (CRP: 19.81 ± 12.56 mg/dl vs. 11.63 ± 11.08 mg/dl, p = 0.001; WBC: 14.7 ± 7.76 g/l vs. 10.88 ± 5.11 g/l, p = 0.005). Both the mean duration of hospitalisation and duration of ICU treatment were longer for patients with a diagnosed CSF infection (p = 0.001; see Table 1). More than half of the patients with associated meningitis required ICU treatment (n = 18; 60%), whereas only 22.78% of the patients without known meningitis (n = 100; 439%) needed to stay in the ICU. The mean duration for an ICU stay was statistically significantly longer statistically for patients diagnosed with meningitis (40.7 ± 22.72 vs 29.54 ± 20.68; p = 0.007). Furthermore, the incidence of epidural abscess and an inflammation of the paravertebral musculature, soft tissue or an abscess of the psoas muscle was statically higher in patients with a CSF infection (n = 18, 60% vs. n = 145, 33.1%; n = 14, 46.67% v.s n = 106, 24.15%; p = 0.003 and 0.006, respectively) (see Table 1).

**Table 1:** Demographics, clinical course and outcome of both patient groups (with and without proven meningitis), reference range/standard values: CSF cell count <5/μl, protein 15-45 mg/dl, glucose 45-80 mg/dl, lactat 1.5-2.0 mmol/l, CRP <0.5 mg/dl, white cells 4.0-9.0 g/l, PCT <0.1 ng/ml.

	patients with an associated meningitis (n = 30; male = 16, female n = 14)	all patients without diagnosed meningitis (n = 439; male n = 281, female n = 158)	
Mean age (yrs)	70.87 ± 8.84 (range 48-88)	67.9 ± 13.1 (range 18-94)	p = 0.490
Mean CSF cell count/μl, *(analysed in 27 patients)*	3375.85 ± 8486.78 (range 32-41550)	-	
Mean value of lactat in CSF (mmol/l) *(analysed in 18 patients)*	6.76 ± 4.37 (range 2.5-18.6)	-	
Mean value of glucose in CSF (mg/dl) *(analysed in 25 patients)*	37.72 ± 34.84 (range 2-169)	-	
Mean protein content in CSF (mg/dl) *(analysed in 25 patients)*	1058.5 ± 1097.9 (range 52-3903)	-	
Mean CRP (mg/dl)/admission	19.81 ± 12.56 (range 2-57) *(analysed in 29 patients)*	11.63 ± 11.08 (range 0.1-64) *(analysed in 434 patients)*	p = 0.001
Mean White blood cell/WBC (g/l)/admission	14.67 ± 7.76 (range 4.1-37.5) *(analysed in 29 patients)*	10.88 ± 5.11 (range 0.8-46.8) *(analysed in 439 patients)*	p = 0.005
Mean Procalcitonin/PCT (ng/ml)	2.16 ± 3.63 (range 0.1-16.3) *(analysed in25 patients)*	1.26 ± 3.31 ng/ml (range 0.1-30,6) *(analysed in156 patients)*	
Mean hospital stay (days)	40.7 ± 22.72 (range 10-95)	29.54 ± 20.68 (range 7-137)	p = 0.007
Number of patients required ICU treatment	n = 18 (60%)	n = 100 (22.78%)	
Duration of required ICU treatment (days)	19.39 ± 23.06 (range 1-94)	15.98 ± 19.23 (range 1-137)	
Distribution of the inflammation	lumbar/lumbosacral n = 16 (53.33%) cervical/cervicothoracic n = 8 (26.67%) thoracic n = 1 (3.33%) thoracolumbar n = 2 (6.67%) multilevel n = 3 (10%)	lumbar/lumbosacral n = 279 (63.55%) cervical/cervicothoracic n = 26 (5.92%) thoracic n = 75 (17.08%) thoracolumbar n = 33 (7.52%) multilevel n = 26 (5.92%)	
epidural abscess	n = 18 (60%)	n = 145 (33.1%)	p = 0.003
inflammation of paravertebral musculature and soft tissue/abscess of the psoas muscle.	n = 14 (46.67%)	n = 106 (24.15%)	p = 0.006
Mean time to diagnosis of meningitis after admission to our department (days)	8 days ±8.32 (range 1-32)	-	
Mean CRP (mg/dl)/discharge	4.38 ± 3.11 (range 0.1-10.9)	5.3 ± 5.56 (range 0.1-43.7)	
Mean White blood cell/WBC (g/l)/discharge	7.6 ± 3.9 (range 3.44 – 23.25)	7.57 ± 3.14 (range 4.4-26.2)	
Mortality % (number of patients who died)	13.33% (n = 4)	7.06% (n = 31)	
Mean ASA	II-III	II-III	
Duration of intravenous antibiotic treatment (days)	20.07 ± 8.58 (Range 14-46)	17.29 ± 9.48 (range 10-90)	p = 0.001
Duration of oral antibiotic treatment (days)	10.78 ± 1.57 range 10-16	10.08 ± 0.64 range 8-16

**Table 2:** Assumed foci of spine infection in patients with associated meningitis.

Assumed foci of spine infection in patients with associated meningitis	n = 30
previous infiltration of the facet joints, subcutaneous or intramuscular infiltration	n = 4
chronic erysipelas/diabetic foot ulcer	n = 5
previous spine surgery	n = 3
previous non-spinal surgery	n = 3
immunosuppression related to drug use	n = 1
systemic tuberculosis	n = 1
parotitis	n = 1
odontogenic infection	n = 1
no focus search remained futile	n = 8
sepsis	n = 3

The most frequent localization of infection was lumbosacral (n = 178, 62.7%) and thoracic (n = 52, 18.3%). Out of 439 patients without meningitis, 31 (7.06%) patients died during the hospital stay or 30 days following due to medical or neurological complications resulting from the underlying spine infection. Successful isolation of the microbial organisms was possible in 284 (64.69%) cases, the most frequent organisms were *Staphylococcus aureus* (n = 106, 24.15%) and Staphylococcus epidermidis (n = 74, 16.86%).

In 15 (3.2%) patients, a CSF examination had been performed with negative results concerning meningitis which could not be verified. The symptoms and reasons requiring a CSF examination were similar to those of the patients who suffered from associated meningitis. Most frequent reasons were delirium, confusion or somnolence (n = 11); fever and epileptic seizures led to diagnosis in one case each. One patient received a (CT)-Myelography; one patient required an external ventricular drainage after suffering from a spontaneous acute subdural hematoma of unclear origin. The mean CSF cell count was 8 ± 8.49/μl.

## 4 Discussion

**Fig. 2:**
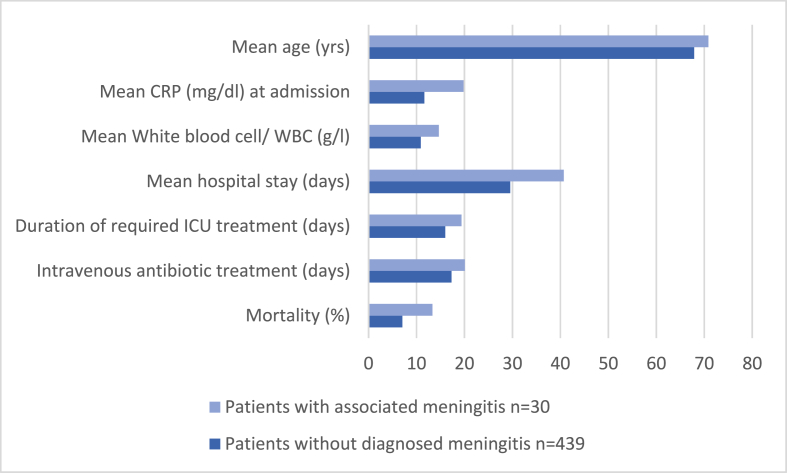
Mean C-reactive protein (CRP) and White blood cell (WBC) count was elevated in all patients at time of admission, whereas they were respectively higher in patients with an associated meningitis: CRP 19.81 ± 12.56 mg/dl vs. 11.63 ± 11.08 mg/dl, (p = 0.001); WBC 14.67 ± 7.76 g/l vs. 10.88 ± 5.11 g/l (p = 0.005).

Considering our data, the number of diagnosed bacterial meningitis due to spondylodiscitis was low. However, the real incidence remains unclear, and the estimated number of unreported cases is suspected to be higher. Out of 469 patients suffering from spondylodiscitis, an associated meningitis was diagnosed in 30 patients (6.4%). At the time of diagnosis, 22 patients presented with somnolence or mental change. Only five of them showed additional neck stiffness, which is one of the most specific symptoms, but none of the patients showed the classic triad characteristic for meningitis. The mortality was relatively high with 13.33% (n = 4). In addition, a CSF examination was conducted in 15 patients (3.2%), yielding negative results with regard to associated infections of the CNS. The symptoms or reasons justifying the examination of the cerebrospinal fluid were similar to those cases where meningitis was found. Except for neck stiffness, there were no specific symptoms indicating meningitis. In fact, the majority of symptoms leading to a CSF examination could also have been caused by one of the common complications of a spondylodiscitis, such as a beginning sepsis. Patients with mild meningitis in particular may be underdiagnosed. Roos et al. described that the recognition of an associated meningitis in patients with an epidural abscess is challenging, but that early diagnosis and rapid treatment are essential to prevent mortality and to decrease neurological sequelae (Roos, 2015). The average duration between admission to our department and diagnosis of meningitis was 8.5 ± 8.2 days. However, it is not clear when exactly the infection passed to the CNS. Furthermore, the diagnosis of spondylodiscitis may be delayed due to nonspecific symptoms especially in elderly patients. In the literature, the average duration between the first symptoms and diagnosis has been reported to be between two and six months (Zarghooni et al., 2012). The mean hospital stay of patients suffering from discitis in the literature varies from 30 to 57 days (Zarghooni et al., 2012). Mean hospital stay of patients suffering from meningitis was 40.7 ± 22.72 days. The mean hospital stay of patients without meningitis was significantly shorter statistically with a mean of 29.54 ± 20.68 days (p = 0.007). The mean time for the duration of intensive care was 19.39 ± 23.06 days for 18 patients suffering from associated meningitis and 15.98 ± 19.23 days for100 patients without a CNS infection. The mean CRP and WBC counts were elevated in all patients at the time of admission, whereas they were respectively higher in patients with an associated meningitis (CRP: 19.81 ± 12.57 mg/dl vs. 11.63 ± 11.8 mg/dl, p = 0.001; WBC: 14.67 ± 7.76 g/l vs. 10.88 ± 5.11 g/l, p = 0.005; Table 1, Fig. 2). The most frequent causative organism was *Staphylococcus aureus* in both groups, which is known as one of the most common organisms in pyogenic spine infection (Sanchez et al., 1999) (Fig. 1). Although due to the unequal patient cohorts, it is obvious that an associated meningitis worsens the outcome and increases the mortality of patients with a spine infection. Out of 439 patients without meningitis, 31 patients died during the hospital stay (7.06%). Cause of death was multi-organ failure due to sepsis in the majority of patients. Out of the 30 patients with an associated meningitis four patients died (13.33%). One patient suffering from cerebral infarction died due to multi-organ failure. An 85-year-old female diagnosed with spondylodiscitis and meningitis by *Mycobacterium tuberculosis* was finally transferred to the palliative care unit. The prognosis of patients with cerebral vasculopathy, which is one of the major complications of bacterial meningitis, is poor. In the study by Pfister et al., only two of 13 patients recovered (Pfister et al., 1992). Bacterial meningitis is the severest form of *Mycobacterium tuberculosis* infection, causing death or severe neurological defects in more than half of those affected (Takahashi et al., 2012; Correa et al., 2001).

The localization of discitis in the cervical spine and the presence of an epidural abscess seems be a potential risk factors for developing an associated meningitis. There was a higher incidence both of cervical discitis (26.67%) and epidural abscess (60%) in the patient group with associated meningitis compared to the control group (5.92% and 33.1%, respectively). Furthermore, the rate of inflammation of the paravertebral musculature, soft tissue and abscess of the psoas muscle was higher (46.67% vs. 24.15%), which may reflect a higher severity of the systemic infection in patients with associated meningitis. The percentage of *Staphylococcus aureus* as the causative organism was also higher in the meningitis group. The physical conditions of the patients were characterized by the ASA risk classification. There were no relevant differences between the two groups (Table 1).

However, due to the retrospective character of this study and the rather unspecific symptoms in the context of an existing spondylodiscitis, the real incidence of associated meningitis remains unclear and the estimated number of unreported cases, especially of cases of mild meningitis, is suspected to be higher. On the other hand, due to the reputation as a spine centre and the nature of being a university hospital, mainly critically ill patients and severe cases of spondylodiscitis are treated in our department. Less complicated cases of spondylodiscitis may be underrepresented.

## 5 Conclusion

Bacterial meningitis due to a spondylodiscitis is a rare but severe condition and is associated with high morbidity and mortality rates. In patients with spine infection who present with an altered state of consciousness, meningitis should be ruled out as soon as possible by CSF examination. The selected antibiotic must be checked for its ability to penetrate the CNS in case of meningitis and the duration of IV therapy must be adjusted if necessary.

## Declaration of competing interest

The authors declare the following financial interests/personal relationships which may be considered as potential competing interests: Berhard Meyer reports a relationship with Ulrich Medical, Medtronic, Depuy Synthes, Brainlab, Spineart, Relievant, Medacta, Icotec, AO Spine, Eurospine and IGASS that includes: consulting or advisory. Maria Wostrack reports a relationship with Icotec, Medacta that includes: consulting or advisory.
